# Predicting Molecular Subtype and Survival of Rhabdomyosarcoma Patients Using Deep Learning of H&E Images: A Report from the Children's Oncology Group

**DOI:** 10.1158/1078-0432.CCR-22-1663

**Published:** 2022-11-08

**Authors:** David Milewski, Hyun Jung, G. Thomas Brown, Yanling Liu, Ben Somerville, Curtis Lisle, Marc Ladanyi, Erin R. Rudzinski, Hyoyoung Choo-Wosoba, Donald A. Barkauskas, Tammy Lo, David Hall, Corinne M. Linardic, Jun S. Wei, Hsien-Chao Chou, Stephen X. Skapek, Rajkumar Venkatramani, Peter K. Bode, Seth M. Steinberg, George Zaki, Igor B. Kuznetsov, Douglas S. Hawkins, Jack F. Shern, Jack Collins, Javed Khan

**Affiliations:** 1 Genetics Branch, NCI, NIH, Bethesda, Maryland.; 2 Advanced Biomedical Computational Science, Frederick National Laboratory for Cancer Research, Frederick, Maryland.; 3 Artificial Intelligence Resource, NCI, NIH, Bethesda, Maryland.; 4 KnowledgeVis, LLC, Altamonte Springs, Florida.; 5 Department of Pathology, Memorial Sloan-Kettering Cancer Center, New York, New York.; 6 Department of Laboratories, Seattle Children's Hospital, Seattle, Washington.; 7 Biostatistics and Data Management Section, Keck School of Medicine of the University of Southern California, Los Angeles, California.; 8 Department of Population and Public Health Sciences, Keck School of Medicine of the University of Southern California, Los Angeles, California.; 9 Children's Oncology Group, Monrovia, California.; 10 Departments of Pediatrics and Pharmacology & Cancer Biology, Duke University School of Medicine, Durham, North Carolina.; 11 Department of Pediatrics, Division of Hematology/Oncology, University of Texas Southwestern Medical Center, Dallas, Texas.; 12 Division of Hematology/Oncology, Texas Children's Cancer Center, Baylor College of Medicine, Houston, Texas.; 13 Institut für Pathologie, Kantonsspital Winterthur, Winterthur, Switzerland.; 14 Cancer Research Technology Program, Frederick National Laboratory for Cancer Research, Frederick, Maryland.; 15 Department of Epidemiology & Biostatistics, School of Public Health, University at Albany, Rensselaer, New York.; 16 Chair of Children's Oncology Group, Department of Pediatrics, Seattle Children's Hospital, Fred Hutchinson Cancer Research Center, University of Washington, Seattle, Washington.; 17 Pediatric Oncology Branch, Center for Cancer Research, NIH, Bethesda, Maryland.

## Abstract

**Purpose:**

Rhabdomyosarcoma (RMS) is an aggressive soft-tissue sarcoma, which primarily occurs in children and young adults. We previously reported specific genomic alterations in RMS, which strongly correlated with survival; however, predicting these mutations or high-risk disease at diagnosis remains a significant challenge. In this study, we utilized convolutional neural networks (CNN) to learn histologic features associated with driver mutations and outcome using hematoxylin and eosin (H&E) images of RMS.

**Experimental Design:**

Digital whole slide H&E images were collected from clinically annotated diagnostic tumor samples from 321 patients with RMS enrolled in Children's Oncology Group (COG) trials (1998–2017). Patches were extracted and fed into deep learning CNNs to learn features associated with mutations and relative event-free survival risk. The performance of the trained models was evaluated against independent test sample data (*n* = 136) or holdout test data.

**Results:**

The trained CNN could accurately classify alveolar RMS, a high-risk subtype associated with *PAX3/7-FOXO1* fusion genes, with an ROC of 0.85 on an independent test dataset. CNN models trained on mutationally-annotated samples identified tumors with *RAS* pathway with a ROC of 0.67, and high-risk mutations in *MYOD1* or *TP53* with a ROC of 0.97 and 0.63, respectively. Remarkably, CNN models were superior in predicting event-free and overall survival compared with current molecular-clinical risk stratification.

**Conclusions:**

This study demonstrates that high-risk features, including those associated with certain mutations, can be readily identified at diagnosis using deep learning. CNNs are a powerful tool for diagnostic and prognostic prediction of rhabdomyosarcoma, which will be tested in prospective COG clinical trials.

**Translational Relevance**

Pediatric rhabdomyosarcoma (RMS) is an aggressive soft tissue tumor, which is associated with a broad range of pathologic mutations. Multiple studies have identified recurrent mutations and chromosomal translocations, which are associated with poor outcome in patients with RMS. Because not all patients are screened for these mutations with molecular testing, there is a need to develop tools which accurately identify these alterations at diagnosis. This study applies deep learning with convolutional neural networks to learn mutation-associated histologic features from H&E images from diagnostic RMS specimens. The deep learning models developed in this study can predict the presence of known high-risk mutations in RMS. We also trained a model, which predicts clinical risk from diagnostic H&E images with performance as well or exceeding current clinical risk stratification. These findings demonstrate that trained A.I. models can identify prognostically relevant features in diagnostic RMS tumor tissue, which could improve current RMS prognostication.

## Introduction

Rhabdomyosarcoma (RMS) is the most common soft tissue tumor in children and adolescent young adults, accounting for 3% of all pediatric cancers. Because clinical staging and risk stratification determines the treatment course of patients, accurately assessing risk at diagnosis is critical for maximizing long-term outcomes. For patients with low-risk RMS, the 5-year survival rate is 70% to 90% whereas high-risk patients have a 5-year survival rate of only 20% to 30% (1). Patients with intermediate-risk RMS, however, have highly variable and unpredictable outcomes and comprise the majority of newly diagnosed patients with RMS (2). Better risk assessment and identification of pathogenic mutations could contribute to better outcomes by personalizing therapy based on an individual's tumor characteristics.

Pediatric RMS is subdivided into three major histomorphologic subtypes: alveolar (ARMS), embryonal (ERMS), or spindle/sclerosing (SSRMS; ref. 3). Survivorship studies previously demonstrated that ARMS histology is associated with a poor outcome relative to ERMS (4, 5). Later molecular studies of ARMS found recurrent chromosome rearrangements t(2;13) or t(1;13), which generate *PAX3–FOXO1* or *PAX7–FOXO1* fusion genes, respectively, and are associated with poor outcome (6–10). These potent fusion oncogenes are present in most ARMS tumors and are referred to collectively as fusion-positive RMS (FP-RMS). Despite the association of FP-RMS with alveolar histology, approximately 10% to 15% of ARMS lack a *PAX3/7–FOXO1* fusion and are transcriptionally similar to ERMS (9, 11, 12). Moreover, patients with fusion-negative RMS (FN-RMS) with alveolar histology have similar outcomes as ERMS (9, 11–13). Large-scale genome profiling of RMS has also identified discrepancies between fusion status and histologic classification (14). Although molecular testing for *PAX3/7–FOXO1* fusions is becoming increasingly common in North America and Europe, not all RMS cases have *FOXO1* fusion molecular testing due to limited available resources, an absence of ARMS histology in the tumor, and other practical reasons such as lack of available resources. Given the prognostic value of fusion status, there is a need to improve the distinction from FP-RMS and FN-RMS tumors at the time of diagnosis.

ERMS is the most common histology and can have a wide range of genetic alterations. The most common driver mutations are in *NRAS*, *HRAS*, *KRAS*, or other genes involved in the *RAS*–*MAPK* pathway and recently these mutations have not been shown to be associated with outcome (15). We also reported that *TP53* mutations are found at diagnosis in 5% to 15% of patients with FN-RMS and are associated with a poor prognosis (14–16). SSRMS, historically, were associated with highly variable outcomes. Following the discovery of MYOD1 activating mutations (p.L122R) in some SSRMS tumors (17), it was realized that tumors harboring a MYOD1 mutation are associated with dismal outcomes whereas MYOD1 wild-type SSRMS have a favorable prognosis (18–21). Despite these emerging genetic risk factors, many FN-RMS cases lack high-risk mutations, yet display a wide range of clinical outcomes. Thus, improvements in prognostication of newly diagnosed patients with RMS requires the detection of high-risk genetic aberrations (e.g., *MYOD1*, *TP53*) with high sensitivity as well as the identification of *de novo* histologic features associated with prognosis.

Predictive models can be developed using artificial intelligence (A.I.) to recognize patterns in pathology images that are associated with clinical or molecular features (22–25). Deep learning with convolutional neural networks (CNN) represents a specific class of machine learning, which is trained by breaking down digital images into small groups of pixels and iteratively identifying salient features using machine learning models. Two recent reports used machine learning to generate predictive models for the major histological subtypes of RMS using hematoxylin and eosin (H&E) images (26, 27). In this study, we report the development of deep learning models that can accurately subclassify RMS fusion status and other clinically relevant genetic alterations from simple diagnostic RMS H&E images. Building on these algorithms, we developed an outcome-based algorithm that predicts event-free survival (EFS) based solely on the H&E images with performance characteristics that match or exceed current methodologies.

## Materials and Methods

### Dataset

All biospecimens were collected on IRB-approved clinical trials or tissue banking studies from COG patients enrolled on ARST0331, ARST0431, D9602, D9803, and D9902. Patient-informed written consent was obtained from each subject or subject's guardian and all studies were conducted in accordance with U.S. Common Rule. H&E pathology slides were de-identified and digitally scanned using an Aperio AT2 (Leica) whole slide scanner at a resolution equivalent to using a 40× microscope objective. Excluding cytology cases and cases with mixed or spindle cell histology, our complete dataset included 88 FP-RMS, 289 FN-RMS, and 3 cases labeled as “not otherwise specified” or NOS.

A tissue microarray (TMA) with 254 cores of 750 μm diameter from 126 additional RMS tumors was constructed from tumors collected at the University Hospital Zurich and at the Kiel Paediatric Tumor Registry. All patients had duplicate cores with the exception of 1 patient who had four cores. The project has been approved by the local ethics committee (KEK-ZH-Nr. 2013–0430) and studies were conducted in accordance with the Declaration of Helsinki. Only several of the patients in the TMA had molecular confirmation of fusion status (*FOXO1* FISH). In lieu of definitive fusion status, we used the ARMS and ERMS clinical annotations which strongly correlate with FP-RMS and FN-RMS, respectively. The TMA contained 250 cores: 201 cores were ERMS and 48 were ARMS as determined by clinical classification of each case. After further review of the TMA by an expert pathologist (E.R. Rudzinski), the number of ERMS cases was revised to 198, the number of ARMS was revised to 52, 1 core was benign stroma containing no evidence of malignancy, and 3 cores that were missing. Overlapping 240 × 240 patches were extracted from each spot and fed into the EfficientNet-B1 architecture. The simple majority prediction probability between ARMS and ERMS were used to classify each core. A consensus A.I. classification for each patient was generated using the geometric means of the class predictions between the two cores. For four patients in which only one tumor core was available, the single core prediction was used for the patient prediction. For the one patient with four duplicate cores, all four cores had the same prediction and was counted as a single consensus A.I. classification. Statistical analyses of the intrapatient A.I. agreement and agreement with pathologist classification are reported as 95% confidence intervals (CI) reported as exact based on Pearson–Klopper method.

Ten whole slides for *MYOD1* mutant cases were generously provided by Dr. Marc Ladanyi (Memorial-Sloan Kettering) and served as an independent validation set. Thirty examples (from 28 slides) of benign normal histology of skin, nerve, and skeletal tissue were requested and received from the NIH Laboratory of Pathology. Slides were obtained from autopsy specimens and de-identified.

### General approach for training and testing deep learning algorithms

**Figure 1.**
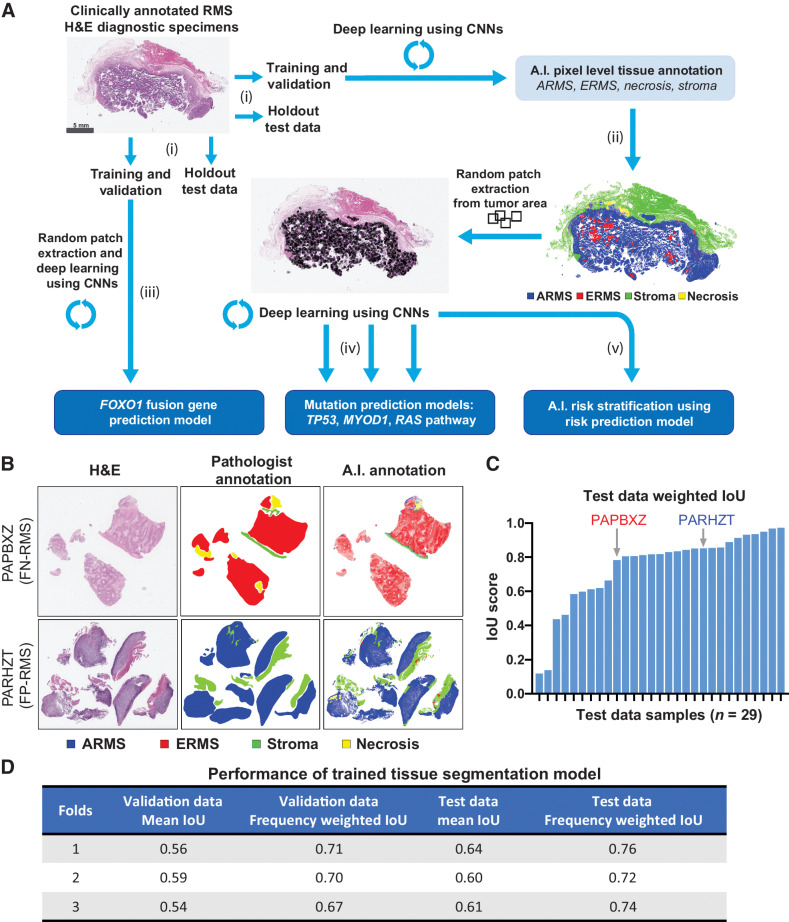
Deep learning of histologic features from RMS tumor tissue. **A,** (i) Samples are randomly selected for training, validation, or holdout test groups with k-fold cross validation. Networks were trained to recognize (ii) basic histological characteristics and (iii) features associated with *FOXO1* fusion status, or (iv) other relevant RMS mutations. (v) A predictive model was also developed to predict disease risk using only an H&E image. **B,** Representative H&E images (left), expert pathologist manual annotation (middle), and pixel-level segmentation results of the A.I. algorithm (right). **C,** Histogram of the weighted IoU scores from holdout test data (*n* = 29). Samples corresponding to **B** are indicated. **D,** Average and weighted intersection over union (IoU) scores of A.I. performance across a 3-fold cross-validation set compared with a pathologist manual annotation.

Our general approach involved extracted patches from digitally scanned whole slide images (WSI) or the TMA at different (10× and 20×) optical magnifications to create the labeled curated dataset. Patches had to contain a majority of tissue or predicted tumor, depending on the model, to be included for training and downstream analyses. Given the class imbalances present in all of our training cohort, we performed patch balancing by oversampling from the minority class and/or performing data augmentation to attain a similar total number of patches between classes. The samples were divided into training, validation, and testing datasets with the majority of samples used for training (28, 29). The validation dataset was utilized for fine-tuning parameters and the network performance was evaluated using holdout testing data. When available, an independent test dataset which was not part of the training cohort was used for evaluating the network performance. The patches were fed into different deep learning architectures [UNet (http://arxiv.org/abs/1505.04597), EfficientNet (http://arxiv.org/abs/1905.11946), and variations of ResNet (http://arxiv.org/abs/1512.03385)] to train the models. We trained deep learning algorithms that could classify FP-RMS versus FN-RMS, segment pixel-level regions of interest (ROI), detect gene mutations (*TP53*, *MYOD1*, and *RAS* pathway), and predict risk for EFS. In models to detect molecular mutations or predict EFS risk, we used the previously trained algorithm to identify areas of RMS (removing predictions of background, stroma, or necrosis) to extract tumor patches for algorithm training as shown in Fig. 1A.

### Tissue segmentation

A total of 78 training samples were used for training a tissue segmentation network with 3-fold cross-validation and were evaluated by an expert RMS pathologist (E.R.R.) to provide ground truth. Out of the 78 samples, 14 or 15 samples (depending on the fold) were set aside as validation samples for fine-tuning the network and an additional 14 or 15 samples were set aside as testing samples for each fold. This experiment was repeated three times and the best performing experiment was reported for segmenting the WSIs. For this experiment, training Fold 1 and Fold 2 contained the same test data (*n* = 14) and the reported performance metrics [mean intersect over union (IoU) and weighted IoU] represent an average of the two folds. We randomly extracted 1,625 patches from FP-RMS WSIs and 1,250 patches from FN-RMS WSIs at 10× optical magnification and each patch size was 384 × 384 pixels. Different numbers of patches were extracted to address class imbalance between FP-RMS and FN-RMS. Otsu thresholding was used to separate tissue foreground and transparent white glass background. Patches that contained less than 50% of the tissue area were not included in the datasets. U-Net architecture with ImageNet pre-trained EfficientNetB4 encoder was selected for segmentation network. We used a combination of weighted categorical cross-entropy and Lovasz-Softmax (https://arxiv.org/abs/1705.08790) loss functions for optimizing the network. A fixed learning rate of 0.0022 was used with RAdam optimizer and batch size of 80 was chosen for training the network. The network was trained for 50 epochs and early termination was applied.

### FN-RMS versus FP-RMS

Our dataset contained 321 patients (69 FP-RMS, 252 FN-RMS, 30 normal). Ten percent of the dataset was set aside for fine-tuning the network (“validation” dataset) and 10% testing dataset was utilized for evaluation of the trained network (“test” dataset). The remaining 80% of the patient samples were used for the training dataset. This experiment was repeated five times for 5-fold cross-validation training. We randomly extracted patches from each WSI at 10× optical magnification and each patch size was 256 × 256 pixels. We performed patch balancing with data augmentation because of class imbalances using the following steps: ARMS: 4,000 patches × 4 [original data + 3 data augmentations (rotate 90°, vertical flip, transpose)] = 16,000 patches per sample; ERMS: 4,400 patches per sample; normal tissues: 5,200 patches × 7 [original data + 6 data augmentations (rotate 90°, rotate 180°, rotate 270°, vertical flip, horizontal flip, transpose)] = 36,400 patches per sample. Patches that did not contain a majority of tissue area were excluded from the training. We used ImageNet pretrained EfficientNetB1 for the training. Multiple instance learning (MIL) loss was utilized under the assumption that not every patch can be clearly classified between FP-RMS and FN-RMS. To select patches with high predictive weight to be included in the training, we used 144 batch size and our MIL loss used the top 70% of each class samples that showed the highest prediction scores. We used a 0.00075 fixed learning rate for 50 epochs and early termination was applied. As an optimizer, Rectified Adam (RAdam; ref. 30) was used for training the network. Among five trained networks, the three best performing networks were selected for inferencing the RMS TMA. Output values from the three networks were averaged to make predictions of the RMS TMA. Normal tissue predictions were discarded and whole tissue predictions were made based on FN-RMS versus FP-RMS probabilities.

### TP53 mutation

We selected 72 FN-RMS samples for training the *TP53* mutation algorithm (Supplementary Table S2). Forty-two patient samples were wild-type samples, which do not contain any gene mutations analyzed (*TP53/MYOD1/RAS* pathway). The rest of the 30 samples were *TP53* mutation positive. Eighteen percent of validation samples were set aside for fine-tuning the network and 18% testing samples were used for trained network evaluation. The remaining patient samples (64%) were used as training data. This experiment was repeated three times for 3-fold cross-validation trainings (Supplementary Fig. S2). We randomly extracted 4,000 patches from wild-type WSIs and 5,600 patches from *TP53*-positive samples (oversampled) to address class imbalance. Patches were extracted from 20× optical magnification WSIs and each patch size was 384 × 384 pixels. Patches were extracted from the segmentation map generated by the tissue segmentation network (from Fig. 1). FP-RMS and FN-RMS ROIs were selected for extracting the patches. We used ImageNet pretrained EfficientNetB3 for the training. Weighted categorical cross-entropy loss was used for optimizing the network. A fixed learning rate of 0.0005 with RAdam optimizer and batch size of 256 was utilized for training. The network was trained for 50 epochs and early termination was applied. *TP53* positive ratio was defined for our training. The ratio was calculated using total number of patches that were predicted as *TP53* divided by total number of patches that were extracted from a sample. As an example, 1,000 patches from a patient were extracted and 300 of the patches were predicted as *TP53*. In this example, the positive ratio becomes 0.3 (300/1,000). We set a threshold at 0.2 to classify *TP53* positive from wild-type samples.

### RAS pathway mutations

Our dataset contained 56 patients with FN-RMS with a mutation in the *RAS* signaling pathway (defined as mutations in *NRAS*, *HRAS*, *KRAS*, *FGFR1, FGFR4*, *NF1*, *PIK3CA*, *FBXW7*, *ERBB2*, *MTOR*, *PTPN11*, *CDKN2A*, *PTEN*, *MET*, *BRAF*, *AKT1*, or *IGF1R*) and we used 49 cases which did not contain any gene-mutations analyzed (*TP53/MYOD1/RAS* pathway) as a control group (Supplementary Table S2). We organized our model training into three random experiments and calculated the metric scores by averaging results from all three experiments. In each experiment, we randomly sample 11 cases from both *RAS* pathway–positive cases and wild-type cases as the hold out test cases and use the rest of cases for 5-fold cross-validation trainings (Supplementary Fig. S3). We randomly extracted 256 × 256 sized patches from predicted ERMS and ARMS regions using previously described RMS tumor segmentation algorithm (from Fig. 1), and we excluded patches with less than 66% tumor areas. We randomly extracted 4,400 patches from wild-type (over-sampled) and 3,700 patches from *RAS*-mutant samples to address class imbalance. We used EfficientNet-B1 with pretrained ImageNet weights as the starting point for transfer learning. We also used a MIL loss function under the hypothesis that not all patches from the *RAS* pathway–positive group might have the mutation. Our MIL loss function skipped samples with low prediction scores based on mean and standard deviation of per-batch sample prediction statistics. For the training, we used a fixed learning rate of 2.5e−6 to train the model for 20 epochs. We saved out the best observed parameters using the ModelCheckpoint callback feature in Keras. We then used our trained models as an ensemble to predict if test patches were *RAS* pathway positive or wild-type. Patches were aggregated on a slide basis to produce a positive prediction score per WSI. We then threshold the positive prediction scores for best separation of the two groups. The positive ratio threshold for predicting RAS pathway mutation is 0.1.

### MYOD1 mutation

We selected 54 patients with FN-RMS for training a *MYOD1* mutation algorithm (Supplementary Table S2). Forty-five cases were selected which did not contain any gene mutations analyzed (*TP53/MYOD1/RAS* pathway) as a control group and nine samples were *MYOD1* mutation positive (all had p.L122R; ref. 15). Thirty-three percent of the validation samples were set aside for fine-tuning the network and the rest of the 67% samples were utilized as training data. This experiment was repeated three times, and holdout test data was not possible due to the low number of *MYOD1*-mutant cases available (Supplementary Fig. S4). We performed patch balancing with data augmentation because of class imbalances using the following steps: Mutant: 4,000 patches × 5 [original data + 4 data augmentations (rotate 90°, 270°, vertical flip, transpose)] = 20,000 patches per sample; wild-type: 4,000 patches per sample. Patches were randomly extracted at 20× optical magnification WSIs and each patch size was 224 × 224 pixels. Patches were extracted from predicted ARMS and ERMS regions from the segmentation map generated by the tissue segmentation network (from Fig. 1). We used ImageNet pretrained Squeeze-and-Excitation ResNet50 (http://arxiv.org/abs/1709.01507) for *MYOD1* mutation detection. Weighted categorical cross-entropy loss was used for optimizing the network. A fixed learning rate of 0.0005 with RAdam optimizer and batch size of 400 patches was utilized for training. The network was trained for 60 epochs and early termination was applied. The positive ratio threshold of 0.043574 was set based on the geometric mean (31) using the validation sample predictions. To evaluate the true performance of this algorithm, we tested an independent dataset composed of 10 additional *MYOD1*-mutant tumors and performed the classification prediction based on the same positive ratio threshold 0.043574.

### Molecular testing

Tumor DNA from the same tissue blocks as part of D9902, ARST08P1, ARST0531, ARST0331, ARST0431, D9803, D9602, and D9802 were extracted and sequenced using a custom panel targeting 39 genes as reported in Shern and colleagues (15).

### EFS analysis

A total of 275 cases classified as FN-RMS by COG were selected for EFS analysis. Eleven cases were excluded because data did not extend beyond 2 years and contained no events recorded. This resulted in 264 cases for training the network (Supplementary Table S3). This represented a rich cohort of patients with diverse biological and molecular characteristics. The deep learning model did not consider any known or A.I.-predicted mutations during training to help identify high-risk patients. Instead, we preferred using EFS as a continuous variable over OS data because EFS tends to be a more sensitive clinical indicator of risk. We used retrospective EFS data to separate the cases into three equal groups of 88 (shortest EFS, intermediate EFS, longest EFS). Cases were randomly and evenly sampled between these groups for training. This prevented any single training iteration from being overweight with high or low EFS cases, which would skew the performance of the model. For each risk category, the cases were divided such that 71 were used for training and 17 used for validation. Patches were extracted using our previously trained algorithm yielding 852k patches for training and 204k patches for validation. The patches were then fed into a deep learning classification architecture (SEResnet-50), where the final layer was altered to be a Cox proportional hazards layer. Training the algorithm was performed using 4-fold cross-validation. We reiterated the 4-fold cross-validation study an additional five times. For each 4-fold cross-validation collection, the independent test dataset was kept static. Each iteration of the 4-fold cross-validation used a different part of the entire dataset from which to determine the independent test dataset. A 20-fold ensemble HR prediction was used for downstream analyses. Because the model makes a continuous “hazard” prediction from −1 to 1, there was a need to define a non-arbitrary cutoff to generate a categorical risk prediction (low, intermediate, high), which could be compared with the clinical risk group assessment.

To find the cutoffs which generated the optimal separation between three groups, an algorithm was written to iterate through many cutoff pairs, at each step separating the cohort into three groups and running log-rank tests. Specifically, three log-rank tests were run at each step: a multivariate test on all three groups, a low-risk versus intermediate-risk test, and an intermediate-risk versus high-risk test. This was to ensure a certain level of separation between all three groups, something which is not guaranteed by a single multivariate test. The specifics of the cutoff search were as follows: The allowed values for the low-intermediate cutoff *C*_L_ fell in the interval [−0.9, 0.5), with a step size of 0.05. For each *C*_L_, the allowed values for the associated intermediate-high cutoff *C*_H_ fell in the interval (*C*_L_, 0.95], again using a step size of 0.05. A subset of possible best cutoffs was saved from the search based on several parameters, and from these the pair with the lowest multivariate log-rank *P* value was chosen. The search parameters were:

– Low-risk vs. Intermediate-risk *P* value must fall below 0.2.
– Intermediate-risk vs. High-risk *P* value must fall below 0.2.
– No group should contain less than 10% of samples.
– No group should contain more than 50% of samples.

Out of the cutoff pairs which fit those criteria, the best multivariate log-rank *P* value was 0.1 (low vs. intermediate) and 0.9 (intermediate vs. high). The resulting A.I.-predicted risk groups were presented as Kaplan–Meier plots and log-rank tests using GraphPad Prism. Patients who had data available beyond 12 years but did not report an event 12 years or less were censored at 12 years (3 patients with EFS data, 4 patients with OS data). This was due to one patient who displayed an unusual event (death at Day 4,835) and was the last datapoint for that risk group.

### Public docker container of deep learning models for segmentation and classification predictions of user-provided RMS H&E images

Browser-based, interactive graphical interfaces for our models have been developed in JavaScript using the Vue.js web interface framework. The models themselves are executed by a serving application written in the Python computer language. This computational system is released as a docker container, which includes the Segmentation, *MYOD1*, and Survivability models (Supplementary Fig. S6; Supplementary Materials and Methods). A docker container is a deployment technique, where an entire application and a minimal runtime system are “bundled together” in a standardized way so the application can be run on a large variety of different computers with different operating systems and various hardware configurations. A docker container of the web interface and models is publicly available for download and testing by any reader at the following address: https://hub.docker.com/repository/docker/curtislisle/rms_infer. By downloading and running the container on their own computer, any interested researchers can test our models locally on their own images. We have also released the open-source code in Python and JavaScript for others in the community to use or extend the deployment system themselves and maximize the benefit of our research to the community. The starting page of the multimodel system displays a mini-application panel for each hosted model (Supplementary Fig. S6). Once the user choses a model to run, the model detail view allows the user to set any configurable parameters and upload an image for processing. If the user does not have a readily available RMS histology image, a preloaded sample image is provided to demonstrate the system. Parameter setting options for the model are provided along the left-hand side of the interface whereas the uploaded image and the calculated results are displayed in the main panel. The interface analyzes user-provided H&E images for calculation of tissue type by percentage on a pixel level (FP-RMS, FN-RMS, stroma, or necrosis) and provides the probability of *MYOD1* mutations and survival risk in relation to our patient cohort. We also developed a server configured to run our models using the same web interface as the docker container but running in the Amazon Cloud. Once our server configuration was complete, a “snapshot” of the disk image for that server was taken. This snapshot is often called an “AMI” (the acronym for Amazon Machine Image). We made this new copy of our server publicly available on the Amazon cloud for any user to upload their own RMS H&E images for predictions using the A.I. models. This AMI is available to any user with a free Amazon Web Services, although there is some minor financial cost to executing a server in the Amazon Cloud. Detailed information how to find and execute our AMI is included in our Supplementary Materials and Methods.

### Data availability

We have deposited the code generated in this study into GitHub at https://github.com/abcsFrederick/RMS_AI. Sequence, metadata, and H&E images will be made available at https://clinomics.ccr.cancer.gov/clinomics/public/viewProjectDetails/25881. Panel sequencing data are available as previously described (dbGAP accession phs000720.v4.p1; ref. 32).

## Results

### Tissue annotation of WSIs using CNNs

We developed a strategy to train models capable of detecting features associated with tumor classification, probability of high-risk mutations, and predict survivability (Fig. 1A). These deep learning models were trained based on a single variable (histology, *FOXO1* fusion, mutation, or EFS) along with the corresponding diagnostic H&E image from the patient. Our first model was trained on RMS H&E images to detect basic histopathologic features including RMS tumor, stroma, and necrosis. An expert pathologist (G.T. Brown) provided detailed annotations as ground truth for ROIs for ARMS, ERMS, stroma, and necrosis, which was used to train a deep learning model with three-fold cross-validation. The trained A.I. algorithm generated a tissue annotation strikingly similar to the expert pathologist's manual annotation (Fig. 1B). Quantification of the percent tissue area showed similar whole slide classification between the pathologist's manual annotation and the annotation predicted from the trained model (Supplementary Table S1). To determine the degree of overlap between pathologist and A.I. tissue annotation, we performed an IoU analysis using holdout test data. There was a high degree of overlap as measured by weighted IoU scores which accounts for both pixel-level overlap and class abundance in individual samples (Fig. 1C). The performance was similar between the three folds with a mean IoU of 0.62 and a mean weighted IoU of 0.74 on the holdout test data (Fig. 1D). This pixel-level overlap was despite the broader tissue classifications by the pathologist compared with the A.I. algorithm providing truly pixel-level classification (Fig. 1B).

### Deep learning of *FOXO1* fusion status from diagnostic H&E images

**Figure 2.**
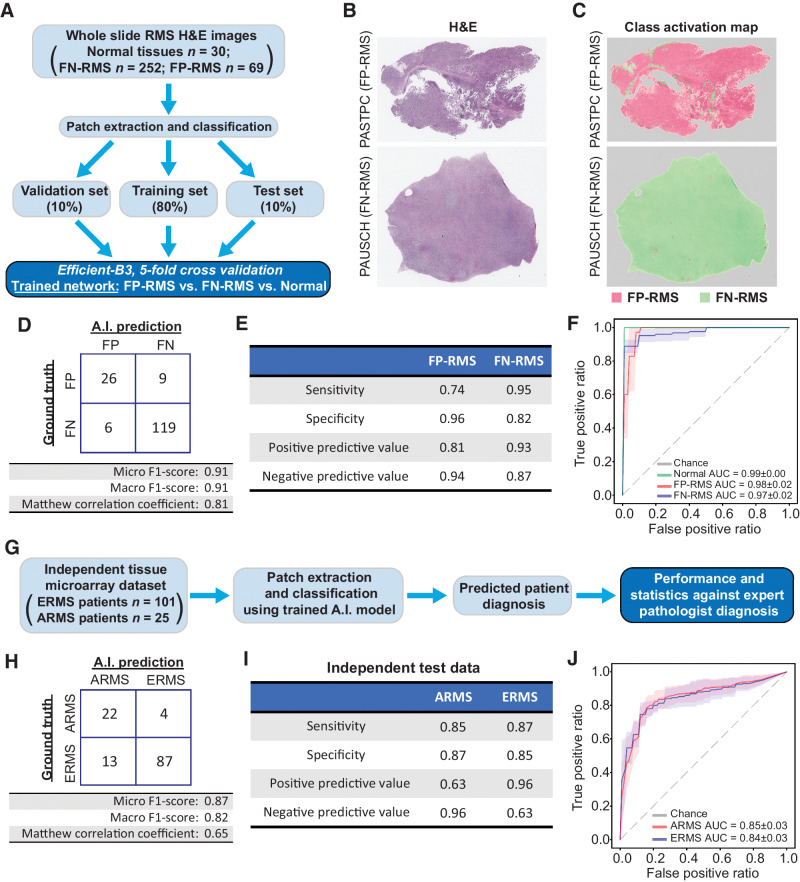
Fusion status prediction from H&E images. **A,** Workflow for deep learning of normal tissue, FN-RMS, and FP-RMS. **B** and **C,** Representative (**B**) H&E images and (**C**) class activation maps as predicted by the trained model. **D,** Confusion matrix for predictions on a test dataset. Micro F1, Macro F1, and Matthew's correlation coefficient shown below. **E,** Performance statistics for FP-RMS and FN-RMS prediction. Note: Normal tissues (*n* = 15) were classified with 100% sensitivity and 100% specificity and are included in performance metric calculations. **F,** Average ROC curve for FP-RMS vs. FN-RMS vs. normal predictions using holdout test data. **G,** Workflow for testing the trained algorithm against an independent RMS TMA dataset. **H,** Confusion matrix for predictions on a test dataset. Micro F1, Macro F1, and Matthew's correlation coefficient shown below. **I,** Performance statistics for FP-RMS and FN-RMS predictions on an independent dataset. **J,** Average ROC curve for FP-RMS vs. FN-RMS using an independent RMS tissue dataset.

We next trained a convolutional neural network to identify normal tissue and distinguish FP-RMS from FN-RMS without manual ROI selection by a pathologist. Diagnostic whole slide H&E images from patients with RMS (*n* = 321) were used for training the model with 5-fold cross-validation (Fig. 2A). The trained predictive model was able to distinguish FN-RMS and FP-RMS tumor tissues as shown in representative class activation maps (Fig. 2B and C). We tested the model's performance on holdout test data and observed an impressive ability to predict fusion status when evaluated with multiple statistical metrics including a high Matthew's correlation coefficient (33) of 0.81 (Fig. 2D and E). The algorithm was also 100% sensitive and specific for normal skin, muscle, and nerve tissue taken from autopsy specimens. The ROC AUC was 0.98 and 0.97 for identifying FP-RMS and FN-RMS tissues, respectively (Fig. 2F).

To further evaluate the performance of our algorithm, we used a clinically annotated RMS TMA as an independent test set containing 254 spots from 126 patient tumors (Fig. 2G). Because molecular validation of fusion status was not available for these samples, we used the histologic clinical classifiers ARMS (likely FP-RMS) and ERMS (likely FN-RMS) to define the groups. The majority of patients (122 of 126 patients) had duplicate cores in the array, which allowed us to assess intrapatient heterogeneity as well as patient-level classification performance. For the 122 patients with duplicate cores, the A.I. classification matched 89% (95% CI, 82%–94%) of the time. Of these samples with matched A.I. predictions, 87% (95% CI, 79%–93%) of them ultimately agreed with the pathologist classification (Supplementary Figs. S1A and S1B). When two A.I. predictions were mismatched for the same patient (*n* = 13), the geometric mean that was used to generate the A.I. consensus classification, was ultimately correct for 10 of 13 patients (77%; 95% CI, 46%–95%; Supplementary Fig. S1C). In total, 95 of the 122 patients (78%; 95% CI, 69%–85%) with duplicate cores had a classification in agreement with the expert pathologist classification (Supplementary Fig. S1D). The performance of the algorithm was evaluated using multiple performance metrics, which showed strong predictive power of our algorithm, particularly for patients with ERMS (FN-RMS; Fig. 2H–J).

### *TP53* mutations can be predicted from tumors with high VAF

**Figure 3.**
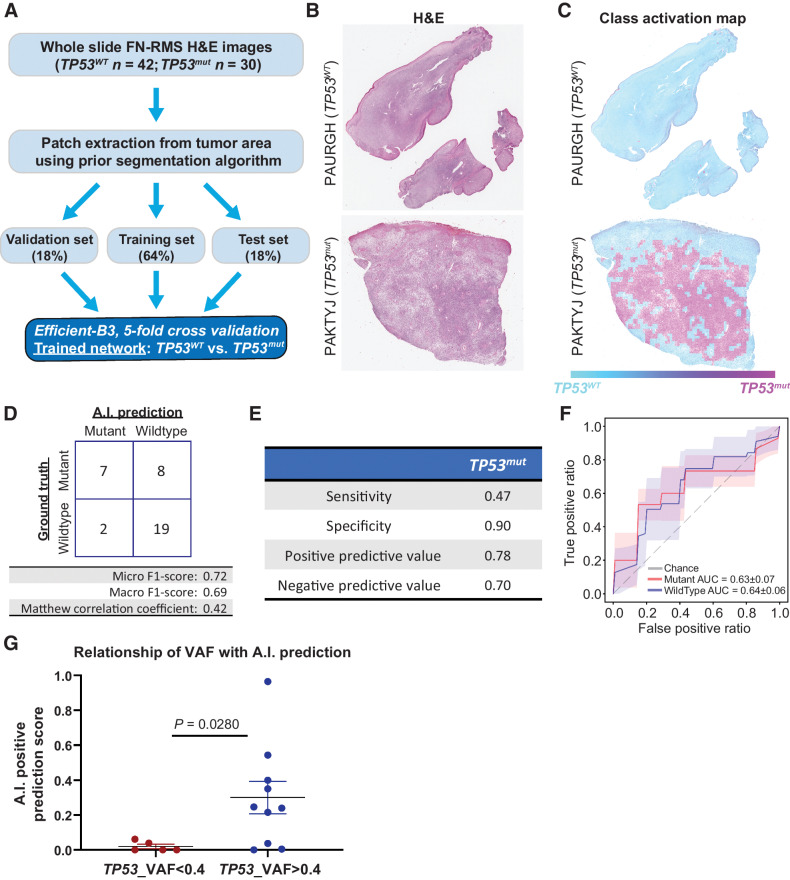
*TP53* gene mutation prediction from H&E images. **A,** Workflow for deep learning of *TP53* mutations from FN-RMS WSIs. **B** and **C,** Representative (**B**) H&E images and (**C**) class activation maps of a TP53 wild-type tumor and a tumor with a TP53 p.P278T mutation (VAF = 0.474). **D,** Confusion matrix for predictions on a test dataset. Micro F1, Macro F1, and Matthew's correlation coefficient shown below. **E,** Performance statistics for *TP53* mutation prediction. **F,** Average ROC curve for *TP53* mutation prediction using holdout test data. **G,** Dot plot of *TP53* mutant samples (*n* = 15) showing relationship between *TP53* mutation VAF and A.I. positive prediction probability. Statistical analysis was performed using the Mann–Whitney *U* test.

A similar deep learning algorithm was trained to identify mutations in *TP53* which are observed in 5% to 15% of patients with FN-RMS and are associated with reduced survival (14–16). Among the patients with FN-RMS in our cohort, there were 34 cases with a *TP53* mutation and 287 *TP53* wild-type cases (Supplementary Table S2; refs. 15, 29). *TP53* wild-type and *TP53* mutant samples were randomly selected and distributed into validation, training, and holdout test datasets (Supplementary Fig. S2). We extracted patches from the area predicted to contain tumor using our previously trained segmentation model from Fig. 1. These patches were fed into an Efficientnet-B3 CNN architecture to train an algorithm that was able to extract features associated with *TP53* mutations (Fig. 3A–C). Using 3-fold cross-validation on holdout test data, the resulting algorithm achieved a specificity of 90% with an ROC AUC of 0.63 for identifying *TP53* mutant tumors (Fig. 3D–F). We noted the low sensitivity of our model with 7 of 15 (47%) of *TP53* mutant tumors being correctly identified. We considered intratumoral heterogeneity with respect to the *TP53* mutation as a potential confounding variable. When distinguishing samples using an arbitrary *TP53* VAF cut-off of 0.40, we found a significant correlation between *TP53* VAF and the mutation prediction probability (Fig. 3G).

### *RAS* pathway gene mutations can be predicted using deep learning

**Figure 4.**
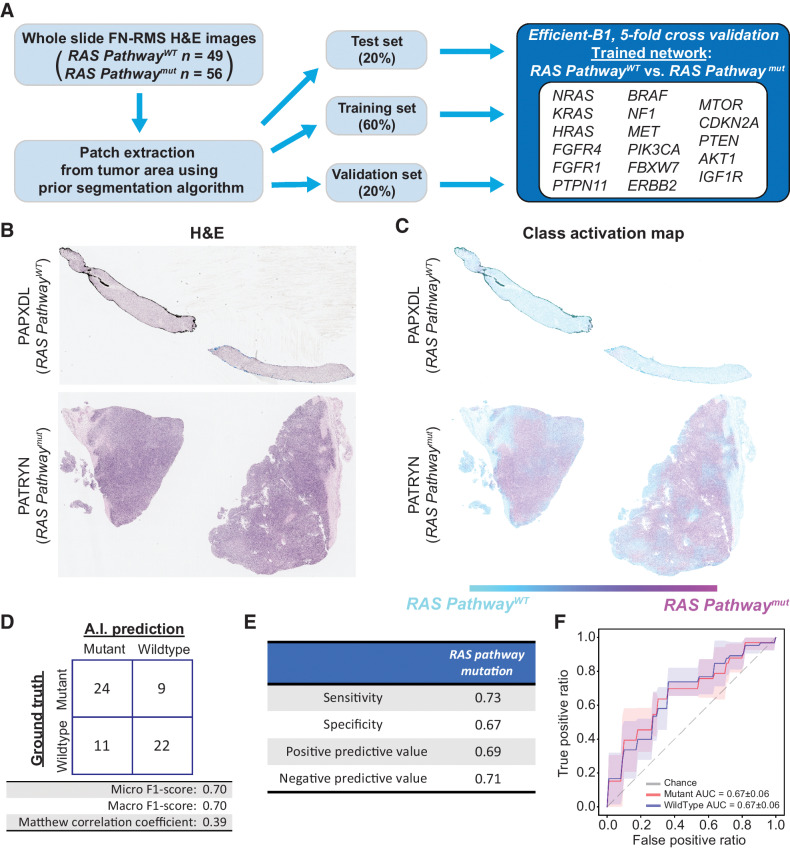
Prediction of RAS pathway mutations using a trained CNN. **A,** Workflow for deep learning of RAS pathway mutations from FN-RMS WSIs. **B** and **C,** Representative (**B**) H&E images and (**C**) class activation maps of a RAS pathway wild-type tumor and a tumor with a KRAS p.G12C mutation (VAF = 0.659). **D,** Confusion matrix for predictions on a test dataset. Micro F1, Macro F1, and Matthew's correlation coefficient shown below. **E,** Statistics for confusion matrix. **F,** Average ROC curve from holdout test data.

We next trained a model to predict mutations in *RAS* pathway genes from WSIs of FN-RMS cases. First, the tumor area was defined using our previously trained segmentation model from Fig. 1. Patches were randomly extracted and fed into an Efficientnet-B1 CNN architecture to train an algorithm that was able to extract features associated with *RAS–MAPK* pathway mutations (17 gene panel; Fig. 4A; Supplementary Table S2; ref. 15). FN-RMS cases which were wild-type (*n* = 49 patients) or mutant in a *RAS–MAPK* pathway gene (*n* = 56 patients) were randomly partitioned for training with cross-validation or for holdout test data (Fig. 4A; Supplementary Table S2). We repeated this across three random experiments with 5-fold cross-validation each and used the average of the three experiments for testing performance (Supplementary Fig. S3). The trained deep learning model was able to distinguish *RAS* wild-type tumors from those with a mutation in a *RAS* pathway gene. As an example, a patient with a KRAS p.G12C mutation (sample PATRYN) was correctly identified as having a *RAS* pathway mutation (Fig. 4B and C). The three experiments showed a range of accuracy from 68% to 77% with a final average accuracy of 70%. Furthermore, the model displayed good sensitivity (73%) and specificity (67%) against holdout test data with a ROC AUC of 0.67 for classifying *RAS* mutant tumors (Fig. 4D–F). This *RAS* pathway algorithm is distinct from other deep learning methods in that it detects features associated with multiple gene mutations, which are known to contribute to an oncogenic signaling pathway in FN-RMS.

### Prediction of MYOD1 p.L122R mutations from diagnostic H&E images

**Figure 5.**
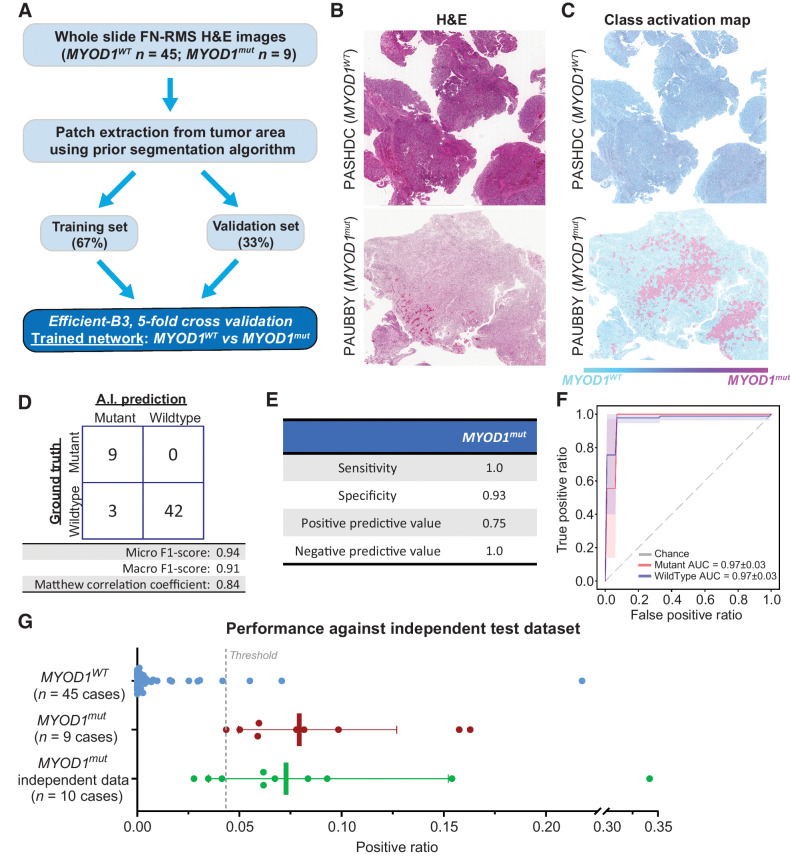
*MYOD1* gene mutation prediction from H&E images. **A,** Workflow for deep learning of *MYOD1* mutations from FN-RMS WSIs. **B** and **C,** Representative (**B**) H&E images and (**C**) class activation maps of a MYOD1 wild-type tumor and a tumor with a MYOD1 p.L122R mutation (VAF = 0.919). **D,** Confusion matrix for predictions on a test dataset. Micro F1, Macro F1, and Matthew's correlation coefficient shown below. **E,** Performance statistics for *MYOD1* mutation prediction. **F,** Average ROC curve for *MYOD1* mutation prediction using validation data. **G,** Plot of *MYOD1* mutation positive ratio thresholds with test datasets (blue = known *MYOD1* wild-type; red = known *MYOD1* mutant) and independent dataset of known *MYOD1*-mutant tumors *n* = 10 (green) shown as geometric mean ± 1 geometric SD.

MYOD1 hotspot mutations (p.L122R) occur in ∼3% of FN-RMS and have been reported by our group and others to be nearly uniformly fatal (15). We next tested whether we could develop an algorithm that could detect *MYOD1* mutations. The *MYOD1* mutant cases used for training our algorithm had cases with SSRMS histology or dense ERMS histology (cases described in ref. 15) and represent the spectrum of histologies that have been associated with *MYOD1* mutations. We used 9 patients with a *MYOD1* mutation and randomly selected 45 *MYOD1* wild-type FN-RMS cases as a control group (Supplementary Fig. S4; Supplementary Table S2; ref. 15). We again used our segmentation algorithm from Fig. 1 to extract patches from the predicted tumor area and feed them into a deep learning neural network (Fig. 5A–C). Using a 3-fold cross validation on holdout test data, we achieved a sensitivity and specificity of 100% and 93%, respectively, for *MYOD1* mutation with an ROC AUC of 0.97 (Fig. 5D–F). We next evaluated the sensitivity of our trained models in predicting *MYOD1* mutations against an independent test dataset of WSIs from *MYOD1*-mutant RMS cases (*n* = 10). Our model correctly classified 7 of 10 cases as high probability for a *MYOD1* mutation, demonstrating excellent sensitivity of the trained model (Fig. 5G). Of note, all three false-negative cases had high positive predictive scores but fell just below our classification threshold (Fig. 5G).

### Patient with FN-RMS disease risk can be predicted from diagnostic H&E images alone

**Figure 6.**
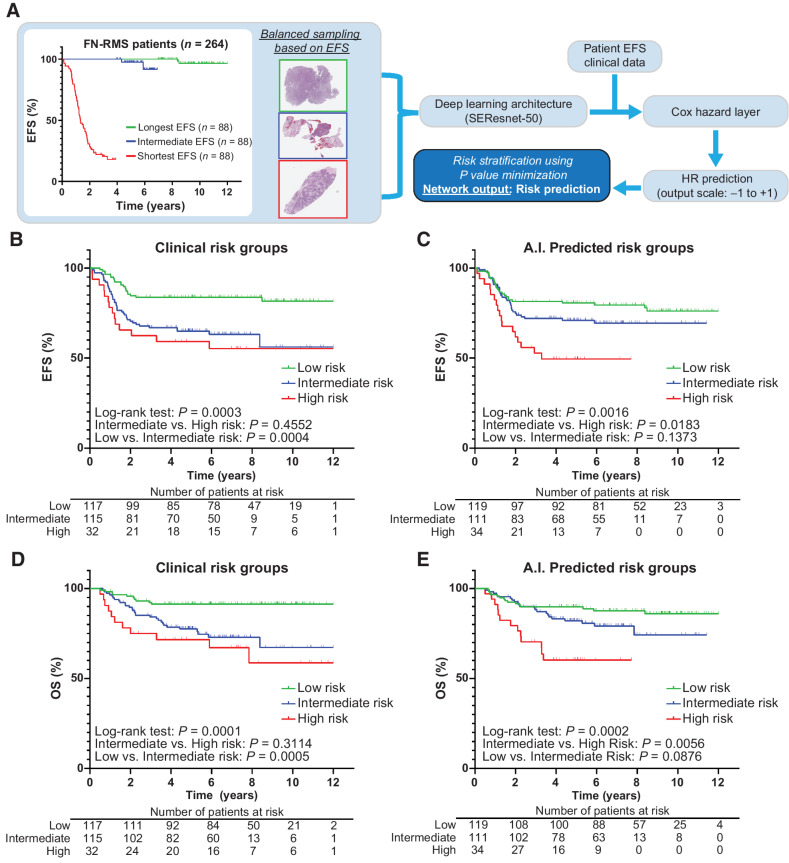
Deep learning algorithms can predict event-free and overall survival from H&E histology alone. **A,** Schematic for training a convolutional neural network for survival. Patients with FN-RMS (*n* = 264) were partitioned into three groups based on EFS length to ensure even sampling and fed into a deep learning architecture with a Cox proportional hazard layer to assess risk. **B,** Kaplan–Meier curves for EFS based on COG clinical risk group assessment. **C,** Kaplan–Meier curves for event-free survival based on A.I. hazard prediction. **D,** Kaplan–Meier curves for OS based on COG clinical risk group assessment. **E,** Kaplan–Meier curves for OS based on the same A.I.-predicted risk grouping as in **C**.

More than half of FN-RMS cases are classified as intermediate risk with current risk stratification guidelines despite a wide range of outcomes from 50% to 90% 5-year overall survival (OS; ref. 2). We investigated whether a deep learning survival model could predict risk of patients with FN-RMS from simple, diagnostic H&E images, as a tool to complement existing clinicopathologic risk stratification. From our dataset, we selected *n* = 275 FN-RMS cases from which we excluded 11 because data was not collected beyond 2 years, or had no events recorded, which likely represented patients that were lost to follow up or dropped out of the study due to reasons unrelated to disease-specific events. The remaining *n* = 264 cases were divided equally into shortest EFS, intermediate EFS, and longest EFS groups for training (Fig. 6A; Supplementary Table S3). Equal numbers of cases were sampled from each group and sent through a deep learning classification architecture which, unlike our previous algorithm architectures, contained an additional Cox proportional hazards layer (Fig. 6A). The output of the algorithm was a “hazard” prediction value (scaled −1 to +1), which provides a relative assessment of risk. We used *P* value minimization logic to define cutoffs of the predicted “hazard” values to define A.I. risk groups (Supplementary Table S3). We analyzed the COG clinical risk group of our patient cohort and found that whereas patients classified as low-risk were strongly associated with long EFS, there was little difference in EFS between patients classified as intermediate- and high-risk (Fig. 6B). Kaplan–Meier analysis with our A.I.-predicted risk groups performed similarly well in identifying low-risk patients (Fig. 6C). Remarkably, our A.I. model provided a significant improvement in distinguishing patients with high-risk from intermediate-risk RMS (*P* = 0.0183) compared with clinical risk prediction (*P* = 0.4552; Fig. 6B and C). The A.I. risk stratification based on EFS training data also demonstrated a superior prognostic ability with overall survival (Fig. 6D and E). Clinical risk grouping provided only marginal separation between intermediate- and high-risk group outcomes (*P* = 0.3114), whereas the A.I.-predicted risk groups clearly identified patients who had the poorest survival (*P* = 0.0056; Fig.6D and E). High-risk features predicted by our model were not associated with enrichment of samples with high-risk mutations such as *MYOD1* or *TP53* (Supplementary Figs. S5A and S5B). Finally, we developed an executable docker container for the algorithms developed in this study (Supplementary Fig. S6). Users can upload RMS H&E images, which can be analyzed by our trained models to provide tissue segmentation statistics, *MYOD1* mutation predictions and relative risk predictions.

## Discussion

We here present the first study to predict mutations in RMS using deep learning of whole slide H&E images from diagnostic specimens. Initial diagnosis and risk stratification is arguably the most critical point of care for patients with cancer since it determines the clinical management and long-term risk to disease recurrence. Along with clinical indicators, histology has historically been used for RMS risk stratification with alveolar histology correlating with poor prognosis. Multiple retrospective and prospective studies have since shown that histologic patterns in RMS are largely surrogate markers for specific mutations and that these mutations, not necessarily histology, are highly predictive of outcomes (2, 9–12, 15, 16, 19, 21, 32, 34). Therefore, we focused our study on developing robust deep learning models to predict the presence or absence of known high-risk mutations from diagnostic whole slide H&E images.

Our CNNs accurately identified the most prognostically relevant genomic alterations in RMS (*PAX3/7–FOXO1* fusion and mutations in *MYOD1*, *TP53*). *TP53* mutations are present at diagnosis in 5% to 15% of patients with FN-RMS (14–16) and although anaplasia is associated with *TP53* mutations in other tumors, only 60% to 70% of *TP53*-mutant RMS display focal or diffuse anaplasia (32, 35). Moreover, only 24% of anaplastic RMS contains a *TP53* mutation (32). Beyond histologic variability, intratumoral genetic heterogeneity may also be confounding as recently acquired *TP53* mutations may be nonuniformly distributed within the tumor. The variability of histologic features associated with *TP53* mutations also appeared to affect the sensitivity of our predictive model in which only tumors with a VAF >0.4 were correctly identified. Future studies incorporating mutation VAF or spatial information using *in situ* mutation detection methods (36–38) could improve CNN model training by providing the distribution of mutations such as *TP53*.

Like *TP53* mutations, *MYOD1*-mutant tumors have a characteristic histology (18, 19, 21) but definitively identifying *MYOD1* mutations based on histology alone is imperfect. A recent study found 10 of 21 spindle or sclerosing RMS contained a *MYOD1* mutation with the sclerosing component correlating more strongly with *MYOD1* mutations than the spindle cell histology (21). These distinctions between histology and mutations are clinically important as anaplasia or spindle cell morphology in the absence of *TP53* or *MYOD1* mutations, respectively, are not associated with inferior outcomes (32, 34, 39). In current clinical practice, selection for molecular testing is biased toward tumors displaying the mutation-associated histology. Tumors with *TP53* or *MYOD1* mutations but lacking the classic histologic patterns might not be sent for molecular testing, leading to potential undertreatment of the patient. We propose that deep learning algorithms can augment the assessment by a pathologist to rapidly and accurately identify tumors with a high probability of *MYOD1* mutation at diagnosis, which could then be confirmed with molecular testing. Ultimately, as more tumors are correctly identified that harbor these mutations, it will allow for improved prognostication and opportunities for targeted therapies with drugs that show activity in *RAS* pathway or *MYOD1*-mutant tumors (40–42).

The most significant aspect of our study was using deep learning to detect features related to outcome (EFS) using only a single diagnostic H&E image. We found this algorithm performed as well, if not better, than the respective clinical risk grouping which considers clinical factors like tumor size and site, staging, and patient age. The predicted frequencies of low-, intermediate-, and high-risk patients were strikingly similar to the historic distribution for RMS patients. Our model classified 13% of patients with FN-RMS as high risk, which is unlike other predictive models which tend to overclassify RMS patients as being high risk (27). Other machine learning studies use multimodal approaches and incorporate clinical information, known molecular characteristics, and/or other imaging modalities into their predictive models (43–46). This approach can have high predictive power but requires large, curated datasets to train accurate models. Since this is currently not feasible for pediatric tumors like RMS, we trained unimodal neural networks to identify features from a single variable that we know has significant prognostic value.

This study attempts to address some of the unique challenges that persist for diagnosing pediatric malignancies. The foremost hurdles include their low incidence, and a unique biology that is distinct from adult equivalent malignancies (47). Furthermore, pediatric cancers tend to have fewer somatic mutations with many of them driven by single chromosomal translocation events such as the *PAX3–FOXO1* fusion in RMS or *EWSR1* fusions in various soft tissue and bone tumors (14, 48–50). As a consequence, it takes international collaborations and decades to accumulate sufficient data to identify new molecular, histologic or clinical features that are prognostic but occur in a small minority of cases. Deep learning models like those described in this study can significantly expedite the learning of these features and provide a standardized, quantitative assessment of diagnostic tissue. We envision these predictive models can also play an important role in detecting relevant mutations to then be verified by molecular testing until we enter an era when all tumors have full genomic characterization. This is particularly important for identifying rare mutations or for use in regions of the world where limited resources are available for molecular diagnostics. Finally, risk prediction models like our FN-RMS risk prediction model can improve stratification of heterogeneous patient populations which otherwise are difficult to prognosticate. Future studies will be needed to learn how to best integrate these predictive models into existing risk stratification practices.

In conclusion, deep learning with convolutional neural networks is a powerful tool to identify tumor features, predict the presence of known high-risk genomic alterations, and assess relative risk using diagnostic H&E tissue. We anticipate the methodology used in this study can be applied to other pediatric and adult malignancies and, by making these tools accessible via a containerized deep learning code, deep learning algorithms may be integrated into clinical decision making and facilitate the use of precision medicine. Future studies integrating mutation prediction with hazard prediction models may further improve risk assessment and our models will be evaluated in prospective clinical trials through the COG.

## Supplementary Material

Supplementary Table S1-S3 Supplemental Table S1. Whole slide image tissue segmentation statistics by an expert pathologist and probability prediction using a trained convolutional neural network. Supplemental Table S2. Clinical and molecular characteristics of FN-RMS samples used for training models for mutation prediction. Yellow boxes indicate genes included in defining the RAS pathway. Supplemental Table S3. Clinical information with COG risk stratification of FN-RMS samples used for training a prognostication predictive CNN.

supplementary table legend1 supplementary table legend

Supplementary_Methods1 Supplemental Description of the Rhabdomyosarcoma Web-based Application

Figure S1 Supplemental Figure S1. Performance of A.I. model in classifying multiple samples from the same patient.

Figure S2 Supplemental Figure S2. Sample partitioning for training and testing a TP53 mutation predictive model using K-fold cross-validation.

Figure S3 Supplemental Figure S3. Sample partitioning for training and testing a RAS pathway mutation predictive model using K-fold cross-validation. Three independent experiments were trained on a random selection of samples for training, validation and testing.

Figure S4 Supplemental Figure S4. Sample partitioning for training a MYOD1 mutation predictive model using K-fold cross-validation.

Figure S5 Supplemental Figure S5. Frequency of mutations in COG and A.I. designated risk groups.

Figure S6 Supplemental Figure S6. Graphical User Interface for tissue segmentation, MYOD1 mutation prediction, and risk prediction models.
