# Supplementary material for: Predicting Molecular Subtype and Survival of Rhabdomyosarcoma Patients Using Deep Learning of H&E Images: A Report from the Children's Oncology Group
Source: Clin Cancer Res. 2022 Nov 8;29(2):364–78. doi: 10.1158/1078-0432.CCR-22-1663 (PMC9843436; doi:10.1158/1078-0432.CCR-22-1663)
Supplement: supplementary table legend1 — supplementary table legend [file ccr-22-1663_supplementary_table_legend1_suppls1.docx]

**Supplemental Tables**

**Supplemental Table S1.** Whole slide image tissue segmentation statistics by an expert pathologist and probability prediction using a trained convolutional neural network.

**Supplemental Table S2.** Clinical and molecular characteristics of FN-RMS samples used for training models for mutation prediction. Yellow boxes indicate genes included in defining the RAS pathway.

**Supplemental Table S3.** Clinical information with COG risk stratification of FN-RMS samples used for training a prognostication predictive CNN.
