# Supplementary material for: Predicting Molecular Subtype and Survival of Rhabdomyosarcoma Patients Using Deep Learning of H&E Images: A Report from the Children's Oncology Group
Source: Clin Cancer Res. 2022 Nov 8;29(2):364–78. doi: 10.1158/1078-0432.CCR-22-1663 (PMC9843436; doi:10.1158/1078-0432.CCR-22-1663)
Supplement: Supplementary_Methods1 — Supplemental Description of the Rhabdomyosarcoma Web-based Application [file ccr-22-1663_supplementary_methods1_suppms1.docx]

**Supplemental Description of the Rhabdomyosarcoma Web-based Application**

**Overview**

Our contribution includes an open-source release of the source code for our trained models and also a reference implementation of the RMS models described in our paper implemented as an interactive web server. This web-enabled version is also available as a pre-built docker container. This document describes the use of the web application, its software architecture, and pointers to the available resources.

**Deep Learning Models** **Utilized**

The deep learning models in our work are implemented using the PyTorch deep learning framework. We employed two different classes of deep learning models: segmentation and classification. For segmentation, we used the UNet neural network, and for our classification tasks, we used ResNet networks.

Our segmentation model, which identifies regions containing ARMS, ERMS, necrosis, and stroma in whole slide images, utilizes a standard *UNet* model as implemented by the Segmentation Models package for pytorch^^[[1]](#footnote-2)^^. The UNet employs transfer learning , initialized with weights from prior training using the ImageNet database^^[[2]](#footnote-3)^^, before being trained using images from our cohort.

The MYOD1 classification network uses pre-trained *ResNet50* models. Similar to the segmentation model, the ImageNet dataset was used to provide the pre-trained weights for the model prior to additional training on our cohort of images to predict MYOD1 mutation. The method used in this application is to sample four thousand small patches containing RMS lesions from random locations within the image being analyzed. Our segmentation model (described above) is used to detect the presence of a form of RMS in the patches. These patches are then input to the *ResNet50* model to generate a prediction score. To improve accuracy, our final prediction is taken as the average output value from an ensemble of three separately trained ResNet50 networks.

The Survivability model uses the same approach as the MYOD1 mutation model, by extracting four thousand patches from the source image and generating a prediction score from the neural network output based on these input patches. The classifier model consists of an ensemble of *ResNet18* deep learning networks, each pre-trained on ImageNet. To improve accuracy, our final prediction is calculated as the average output value from an ensemble of twenty separately trained *ResNet18* networks.

**Software Framework for Web Hosting**

To use these models directly requires considerable programming expertise that many pathologists and clinicians don’t possess, so we decided to build a web application that allowed users without programming background to run models on their own data. Because of the integrated models, our web application contains a customized web-server written in python to manage the uploading and storage of user images and the execution of the embedded neural networks.

During the development effort, we tested this web system installed natively on local computer systems, installed natively on an Amazon Cloud EC2 virtual machine instance, and deployed as a docker container. The architecture of the existing system is shown in Figure 1.

The pre-trained models are shown in green. Their execution is managed by an open-source data and job control framework, Girder^^[[3]](#footnote-4)^^, which implements REST endpoints for execution of each model. The user-interface code is written entirely in Javascript and utilizes the Vue.js approach for interactive components.


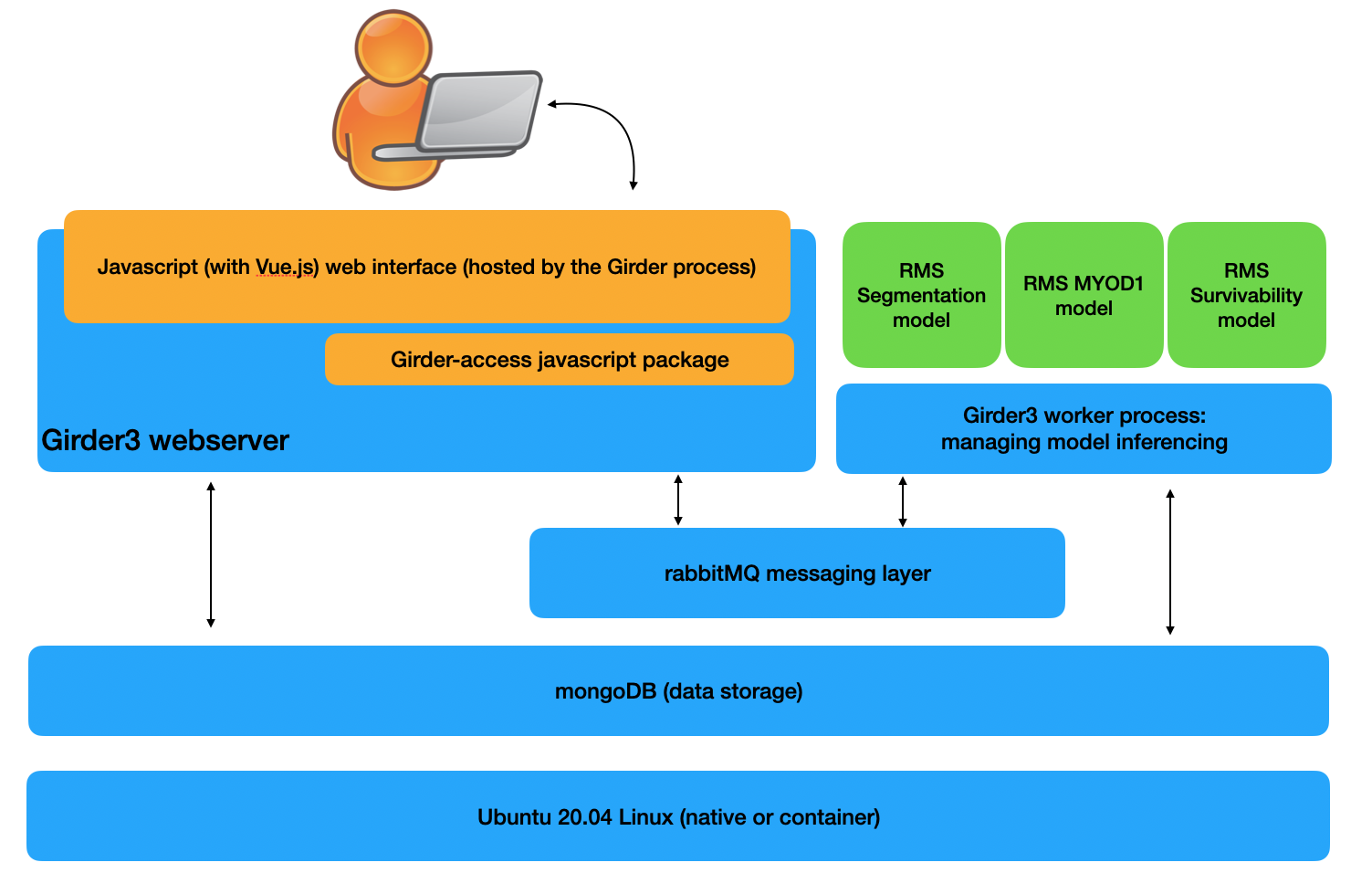


Figure 1 - software design of web application

**System Requirements (GPU and RAM)**

All three of the RMS models (shown in the three green boxes in Figure 1) were initially developed with GPUs in mind for the inferencing because of the computational cost of the models. Then the model code was adopted to allow either GPU or CPU inferencing. CPU-only inferencing is noticeably slower, but easier to host on virtual machines or on the NIH Cloud, for example. The current version of the container uses GPUs when they are available and automatically falls back to CPU inferencing when GPUs are not available.

The segmentation algorithm has to manage a source WSI (whole slide image) and a segmentation WSI in memory simultaneously during execution. For full size WSIs, the segmentation algorithm can approach 128GB of RAM during execution. For small images, 32GB RAM is enough, but for full-scale use, 128GB RAM is recommended for successfully running the inference algorithms. This RAM is only used during execution and is freed immediately after a segmentation process is finished.

**Requirements for use**

Users only need to install our tool and have images to test available in a standard histology file format, such as Aperio (.sys), Hamamatsu (.ndpi), or similar.  Once our tool is installed and running, users can upload images and run predictions using their local browser.  We recommend the use of the Google Chrome browser.  Using this approach, our tool is widely available to the community of users with minimal prerequisites for its execution.

**Software Installation and Execution**

The web-based tool we have developed is available for public download at the following location: https://hub.docker.com/repository/docker/curtislisle/rms_infer.  This tool is a docker container that can be executed on any computer (Windows, Mac, or Linux) that already has the docker tools installed.  Docker tools can be downloaded for no charge from the Docker website (https://www.docker.com/get-started/). Our tool does not require a graphics processor unit (GPU) in the computer, though that will help speedup execution.

As mentioned, the tool is available for download using docker tools on any workstation. This can be accomplished using the commands below.

docker pull curtislisle/rms_infer

docker run -rm -it -p 8080:8080 rms_infer

The first command downloads our tool from Docker’s repository to the user’s local computer. The second command executes the tool on the user’s local computer. After these steps are finished, open a tab using an internet browser to the following URL: <http://localhost:8080> to see the web interface for our application. At this point, please follow the instructions on the loaded web page.

If the user’s computer is equipped with NVIDIA GPUs, our application can be directed to use the GPUs for computation while running our pre-trained deep learning models. To use GPUs during execution, please add the “—gpus all” argument when executing our docker container, as shown below. This will substantially reduce the run time for analyzing uploaded images:

docker run —-gpus all —rm -it -p 8080:8080 rms_infer

Regardless of GPU or CPU hardware, inferencing can take multiple minutes, or up to and even over an hour, depending on the image size and the model selected. Survivability is the most time consuming model of the three models bundled in our web application.

**Amazon Machine Image**

Our team has saved a release of our web interface tool containing the Rhabdomyosarcoma models as an AMI (an Amazon Machine Image) and made this AMI publicly available on the Amazon cloud.  To execute your own version of our RMS interface, go to the AMI Marketplace and search for our AMI under the name “RMS_inference_CCR_2022”. Alternatively, the AMI can be found at the URL:  "[https://us-east-1.console.aws.amazon.com/ec2/home?region=us-east-1#ImageDetails:imageId=ami-012e81874c0478ffa](https://gcc02.safelinks.protection.outlook.com/?url=https%3A%2F%2Fus-east-1.console.aws.amazon.com%2Fec2%2Fhome%3Fregion%3Dus-east-1%23ImageDetails%3AimageId%3Dami-012e81874c0478ffa&data=05%7C01%7Cdavid.milewski%40nih.gov%7Cf6a75ee0c749460a07d708daa27d03a7%7C14b77578977342d58507251ca2dc2b06%7C0%7C0%7C638000955072175998%7CUnknown%7CTWFpbGZsb3d8eyJWIjoiMC4wLjAwMDAiLCJQIjoiV2luMzIiLCJBTiI6Ik1haWwiLCJXVCI6Mn0%3D%7C3000%7C%7C%7C&sdata=DyCGGxYxuc%2F%2FJZvaW6dr5My1dSvjSfXLRe6A8PcIBc8%3D&reserved=0)”.

Once you are viewing the description page of our AMI, which is also known by AMI ID number 012e81874c0478ffa, use the button entitled “Launch Instance from AMI” and follow the steps to create an instance from this AMI.  Our AMI requires a 64GB or larger main disk and an instance size at least comparable to the AWS t2.medium instance class.  The AMI works with either CPU only or GPU-equipped instance types.  If a GPU is available, our models will run faster, but a GPU is not required for execution.  Our RMS segmentation algorithm can require a considerable memory size during execution, and the exact amount of memory needed is a function of the pixel, size of the H&E WSI image used for analysis.  To process large or full-size H&E images, we recommend using an instance with at least 128GB of RAM.

After the computing instance has started, look at the instance page on the AWS Console to find out the public internet address of the running instance, then open a browser tab to the URL of the instance addressed followed by a colon and the port number 8080.  For example, if the IP address of the instance is 100.25.30.212, then the RMS interface would be visible in the web browser at the URL  [http://100.25.30.212:8080](https://gcc02.safelinks.protection.outlook.com/?url=http%3A%2F%2F100.25.30.212%3A8080%2F&data=05%7C01%7Cdavid.milewski%40nih.gov%7Cf6a75ee0c749460a07d708daa27d03a7%7C14b77578977342d58507251ca2dc2b06%7C0%7C0%7C638000955072175998%7CUnknown%7CTWFpbGZsb3d8eyJWIjoiMC4wLjAwMDAiLCJQIjoiV2luMzIiLCJBTiI6Ik1haWwiLCJXVCI6Mn0%3D%7C3000%7C%7C%7C&sdata=zZYg2wk3A7zgzQ%2FpyZ8kPiBc%2FMM4ZrW%2FweeiuaEWe4s%3D&reserved=0).  On the browser interface, several applications (instructions, segmentation, MYOD mutation, and survivability) are displayed.  Then click the application to explore instructional videos or try any of the applications.

Each application allows a demonstration using a pre-loaded image or you can upload your own image.  If our system starts processing an image but isn’t able to successfully complete, the most likely issue is running out of memory on the instance.  If this is the case, select a different type of Amazon instance with more memory available.  For example, an m5.8xlarge instance has 128 GB of Memory available. 

1. Segmentation Models.pytorch online documentation: <https://smp.readthedocs.io/en/latest/> [↑](#footnote-ref-2)
2. Deng et al, “ImageNet: a large-scale hierarchical image database”, https://ieeexplore.ieee.org/document/5206848 [↑](#footnote-ref-3)
3. Girder: a data management platform, https://girder.readthedocs.io/en/latest/ [↑](#footnote-ref-4)
