# Supplementary material for: Predicting Molecular Subtype and Survival of Rhabdomyosarcoma Patients Using Deep Learning of H&E Images: A Report from the Children's Oncology Group
Source: Clin Cancer Res. 2022 Nov 8;29(2):364–78. doi: 10.1158/1078-0432.CCR-22-1663 (PMC9843436; doi:10.1158/1078-0432.CCR-22-1663)
Supplement: Figure S1 — Supplemental Figure S1. Performance of A.I. model in classifying multiple samples from the same patient. [file ccr-22-1663_figure_s1_suppfs1.pdf]

Supplemental Figure S1

a

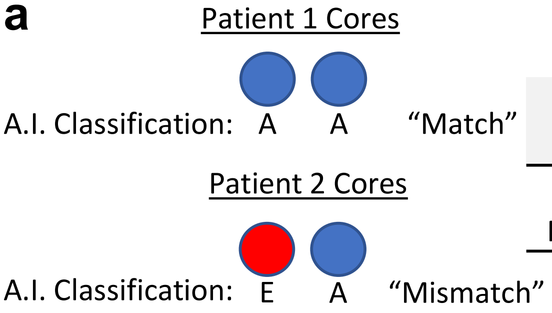

	A.I. classification of duplicate cores		
	Match	Mismatch	Total
Number of patients	109	13	122
Proportion (95% C.I.)	0.89 (0.82, 0.94)	0.11 (0.06, 0.18)	

b

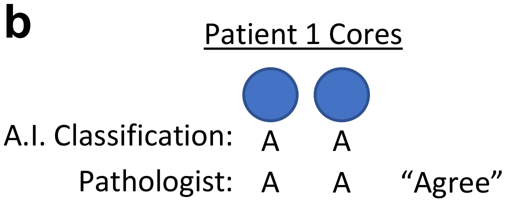

	Agreement between pathologist and consensus A.I. classification for Matched predictions only		
	Agree	Disagree	Total
Number of patients	95	14	109
Proportion (95% C.I.)	0.87 (0.79, 0.93)	0.13 (0.07, 0.21)	

c

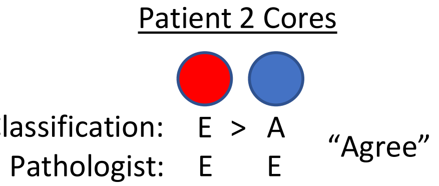

	Agreement between pathologist and consensus A.I. classification for Mismatched predictions only		
	Agree	Disagree	Total
Number of patients	10	3	13
Proportion (95% C.I.)	0.77 (0.46,0.95)	0.23 (0.05, 0.54)	

d

	Final agreement between pathologist and consensus A.I. classifications		
	Agree	Disagree	Total
Number of patients	95	14	122
Proportion (95% C.I.)	0.78 (0.69, 0.85)	0.11 (0.06, 0.19)	

Supplemental Figure S1. Performance of A.I. model in classifying multiple samples from the same patient. An RMS TMA (corresponding to Figure 2g-j) contained two unique tumor cores each for n=122 patients. "A" = ARMS; "E" = ERMS. (**a**) Proportion with 95% confidence interval showing the frequency that the A.I. predictions matched within each patient. (**b**) Proportion with 95% confidence interval showing the frequency that the A.I. predictions that matched (n=109) also agreed with the expert pathologist classification. (**c**) Proportion with 95% confidence interval showing the frequency that the A.I. predictions that were mismatched (n=13) ultimately agreed with the expert pathologist classification by using the geometric mean of the two A.I. sample predictions. (**d**) Proportion with 95% confidence interval showing the total frequency that the ultimate A.I. diagnosis agreed with the pathologist diagnosis for all patients with two unique cores (n=122).
