# Supplementary material for: Predicting Molecular Subtype and Survival of Rhabdomyosarcoma Patients Using Deep Learning of H&E Images: A Report from the Children's Oncology Group
Source: Clin Cancer Res. 2022 Nov 8;29(2):364–78. doi: 10.1158/1078-0432.CCR-22-1663 (PMC9843436; doi:10.1158/1078-0432.CCR-22-1663)
Supplement: Figure S3 — Supplemental Figure S3. Sample partitioning for training and testing a RAS pathway mutation predictive model using K-fold cross-validation. Three independent experiments were trained on a random selection of samples for training, validation and testing. [file ccr-22-1663_figure_s3_suppfs3.pdf]

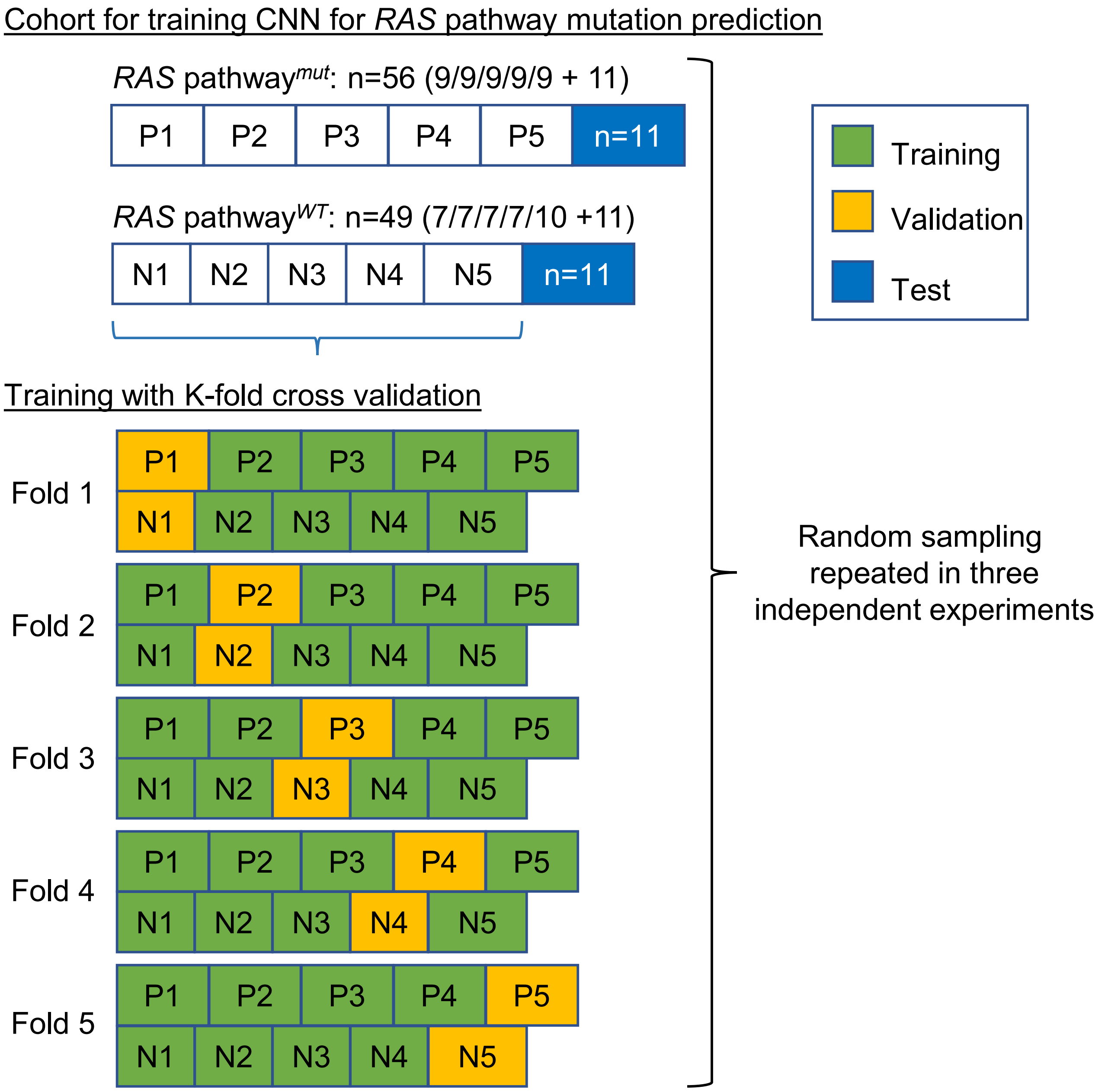

Supplemental Figure S3. Sample partitioning for training and testing a *RAS* pathway mutation predictive model using K-fold cross-validation. Three independent experiments were trained on a random selection of samples for training, validation and testing.
