# Supplementary material for: Predicting Molecular Subtype and Survival of Rhabdomyosarcoma Patients Using Deep Learning of H&E Images: A Report from the Children's Oncology Group
Source: Clin Cancer Res. 2022 Nov 8;29(2):364–78. doi: 10.1158/1078-0432.CCR-22-1663 (PMC9843436; doi:10.1158/1078-0432.CCR-22-1663)
Supplement: Figure S4 — Supplemental Figure S4. Sample partitioning for training a MYOD1 mutation predictive model using K-fold cross-validation. [file ccr-22-1663_figure_s4_suppfs4.pdf]

Supplemental Figure S4

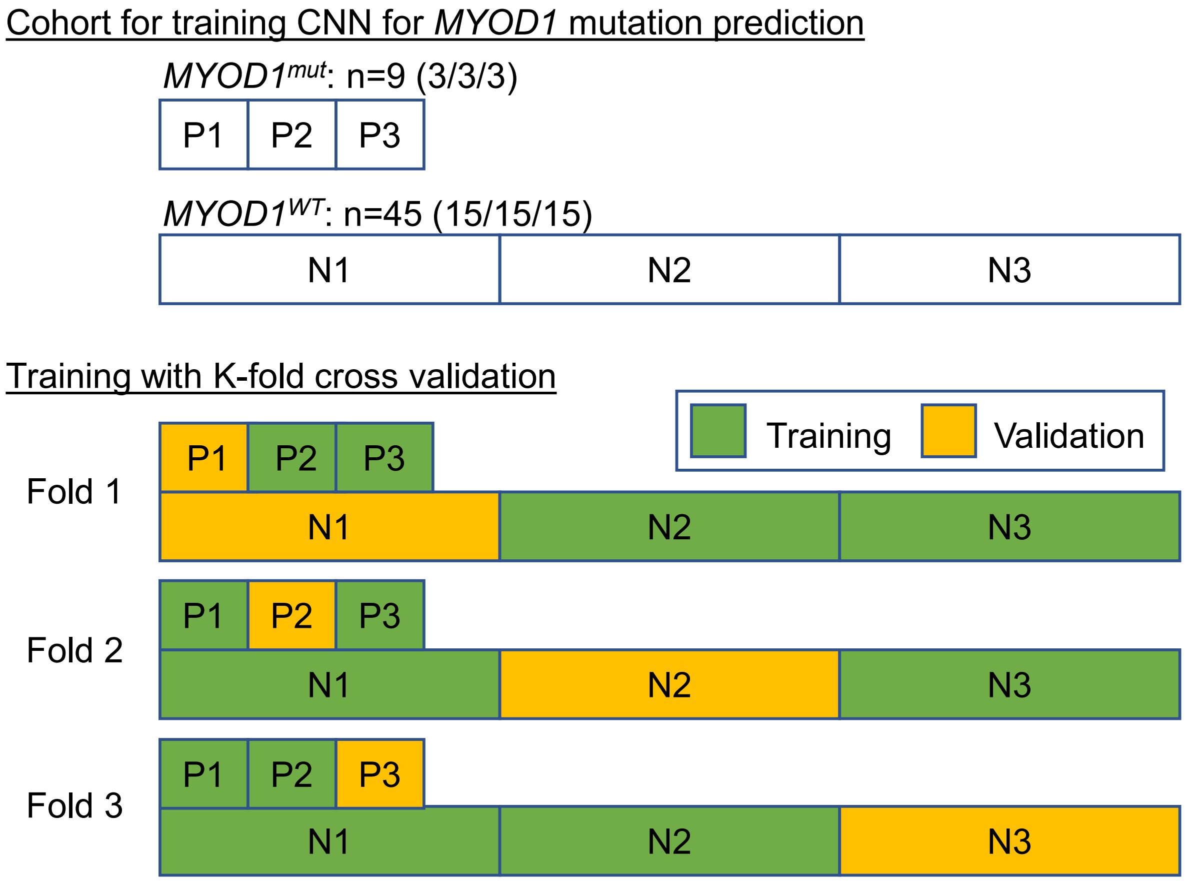

Supplemental Figure S4. Sample partitioning for training a *MYOD1* mutation predictive model using K-fold cross-validation.
