# Supplementary material for: Predicting Molecular Subtype and Survival of Rhabdomyosarcoma Patients Using Deep Learning of H&E Images: A Report from the Children's Oncology Group
Source: Clin Cancer Res. 2022 Nov 8;29(2):364–78. doi: 10.1158/1078-0432.CCR-22-1663 (PMC9843436; doi:10.1158/1078-0432.CCR-22-1663)
Supplement: Figure S5 — Supplemental Figure S5. Frequency of mutations in COG and A.I. designated risk groups. [file ccr-22-1663_figure_s5_suppfs5.pdf]

Supplemental Figure S5

a

Cohort *TP53* mutant (n=33 of 264 cases)

Risk Group	COG Clinical Risk Group Cases (% *TP53*^{mut} cases)	A.I. Predicted Risk Group Cases (% *TP53*^{mut} cases)
Low	14 (42.4%)	15 (45.5%)
Intermediate	13 (39.4%)	13 (39.4%)
High	6 (18.2%)	5 (15.2%)

b

Cohort *MYOD1* mutant (n=11 of 264 cases)

Risk Group	COG Clinical Risk Group Cases (% *MYOD1*^{mut} cases)	A.I. Predicted Risk Group Cases (% *MYOD1*^{mut} cases)
Low	2 (18.2%)	1 (9.1%)
Intermediate	8 (72.7%)	6 (54.6%)
High	1 (9.1%)	4 (36.4%)

Supplemental Figure S5. Frequency of mutations in COG and A.I. designated risk groups. (**a-b**) Distribution of mutations in FN-RMS cases with respect to COG clinical risk group stratification or A.I. risk group stratification from the survivability model. Samples with mutations in (a) *TP53* or (b) *MYOD1* mutations.
