# Supplementary material for: Predicting Molecular Subtype and Survival of Rhabdomyosarcoma Patients Using Deep Learning of H&E Images: A Report from the Children's Oncology Group
Source: Clin Cancer Res. 2022 Nov 8;29(2):364–78. doi: 10.1158/1078-0432.CCR-22-1663 (PMC9843436; doi:10.1158/1078-0432.CCR-22-1663)
Supplement: Figure S6 — Supplemental Figure S6. Graphical User Interface for tissue segmentation, MYOD1 mutation prediction, and risk prediction models. [file ccr-22-1663_figure_s6_suppfs6.pdf]

Supplemental Figure S6

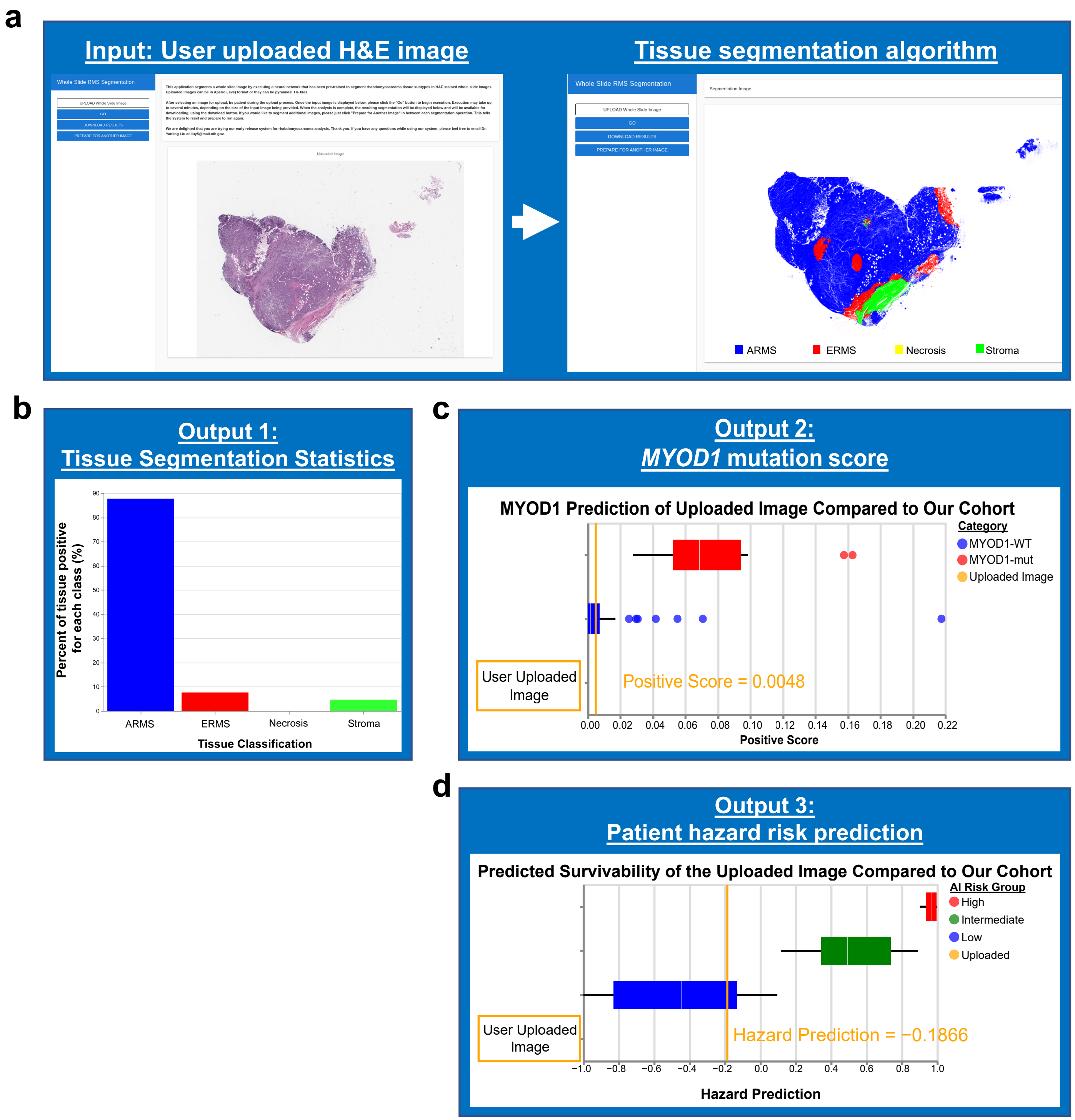

Supplemental Figure S6. Graphical User Interface for tissue segmentation, *MYOD1* mutation prediction, and risk prediction models. (**a**) A containerized docker was developed to allow users to upload H&E images of RMS tissue and perform tissue segmentation to classify regions of tumor. (**b**) Proportions of tissue predicted as ARMS, ERMS, necrosis, and stroma are generated as a bar graph. (**c**) User-provided images can also be analyzed for *MYOD1* mutations using our trained *MYOD1* mutation prediction model. The positive prediction score can be compared with our training cohort of samples that have known MYOD1 mutation status. (**d**) A hazard prediction score (-1 to +1) can be attained using the trained FN-RMS risk prediction model. The hazard prediction score can be compared with A.I. predicted risk grouping for our study cohort data (n=264).
